# Impaired Vitamin D Signaling in Endothelial Cell Leads to an Enhanced Leukocyte-Endothelium Interplay: Implications for Atherosclerosis Development

**DOI:** 10.1371/journal.pone.0136863

**Published:** 2015-08-31

**Authors:** Milica Bozic, Ángeles Álvarez, Carmen de Pablo, Maria-Dolores Sanchez-Niño, Alberto Ortiz, Xavier Dolcet, Mario Encinas, Elvira Fernandez, José Manuel Valdivielso

**Affiliations:** 1 Nephrology Research Department, IRB Lleida, Lleida, Spain; 2 Department of Pharmacology and CIBERehd, University of Valencia, Valencia, Spain; 3 IIS-Fundacion Jimenez Diaz, School of Medicine, UAM and IRSIN, Madrid, Spain; 4 Pathology Group, Pathology and Molecular Genetics Department, Hospital Universitari Arnau de Vilanova, University of Lleida and IRB Lleida, Spain; 5 Department of Experimental Medicine, University of Lleida and IRB Lleida, Lleida, Spain; Max-Delbrück Center for Molecular Medicine (MDC), GERMANY

## Abstract

Endothelial cell activation leading to leukocyte recruitment and adhesion plays an essential role in the initiation and progression of atherosclerosis. Vitamin D has cardioprotective actions, while its deficiency is a risk factor for the progression of cardiovascular damage. Our aim was to assess the role of basal levels of vitamin D receptor (VDR) on the early leukocyte recruitment and related endothelial cell-adhesion-molecule expression, as essential prerequisites for the onset of atherosclerosis. Knockdown of VDR in endothelial cells (shVDR) led to endothelial cell activation, characterized by upregulation of VCAM-1, ICAM-1 and IL-6, decreased peripheral blood mononuclear cell (PBMC) rolling velocity and increased PBMC rolling flux and adhesion to the endothelium. shVDR cells showed decreased IκBα levels and accumulation of p65 in the nucleus compared to shRNA controls. Inhibition of NF-κB activation with super-repressor IκBα blunted all signs of endothelial cell activation caused by downregulation of VDR in endothelial cells. *In vivo*, deletion of VDR led to significantly larger aortic arch and aortic root lesions in apoE^-/-^ mice, with higher macrophage content. apoE^-/-^VDR^-/-^mice showed higher aortic expression of VCAM-1, ICAM-1 and IL-6 when compared to apoE^-/-^VDR^+/+^ mice. Our data demonstrate that lack of VDR signaling in endothelial cells leads to a state of endothelial activation with increased leukocyte-endothelial cell interactions that may contribute to the more severe plaque accumulation observed in apoE^-/-^VDR^-/-^ mice. The results reveal an important role for basal levels of endothelial VDR in limiting endothelial cell inflammation and atherosclerosis.

## Introduction

Vascular endothelium is considered to be a main regulator of blood vessel homeostasis, having not solely a barrier function, but exerting a number of vasoprotective roles. Due to its intrinsic ability to sense humoral and haemodynamic stimuli [1], the endothelium contributes to the local regulation of vascular tone and structure, controls the growth and migration of vascular smooth muscle cells (VSMCs), and regulates the adhesion and extravasation of leukocytes [2, 3]. Hence, impairment of endothelial function is a major pathological mechanism predisposing to diseases like atherosclerosis [4]. Atherosclerosis is characterized by chronic inflammation of the vessel wall initiated by leukocyte recruitment and adhesion to the activated endothelium, and followed by their migration into the subendothelium with subsequent lipid accumulation within macrophages [5]. Elucidating molecules and pathways essential for the preservation of vascular endothelial integrity and function may provide insights that could lead to discovery of efficient therapeutic targets for the treatment of atherosclerotic vascular disease.

Here, we explored the contribution of vitamin D receptor (VDR) in limiting vascular endothelial cell’s (EC) activation and inflammation. VDR is a member of the superfamily of nuclear receptors for steroid/thyroid hormones. VDR has a ubiquitous tissue expression [6] and acts as a ligand-activated transcription factor [7] mediating most of the functions of its preferred ligand, 1,25-dihydroxyvitamin D [1,25(OH)_2_D] [6–9]. The potential actions of 1,25(OH)_2_D expand far beyond its essential roles in calcium homeostasis and bone metabolism [6, 10], reaching highly diverse non-classical actions, such as regulation of cell proliferation and differentiation, immunomodulation, regulation of cytokine production and hormone secretion [9–13]. Furthermore, 1,25(OH)_2_D inhibits various aspects of inflammation [14], a well-known key pathogenic mechanism of atherosclerosis, and protects against myocardial cell hyperthrophy [15] and fibrosis [16], thus emerging as an important player in the protection against cardiovascular disease (CVD) mortality.

A plethora of epidemiological and clinical data suggests a link between vitamin D deficiency and an increased risk of CVD [17–23]. The low vitamin D status may be a contributing factor in the pathogenesis of congestive heart failure [18] and peripheral arterial disease [24], while low levels of serum 25(OH)D are associated with a higher risk of hypertension, diabetes, hyperlipidemia [20] and myocardial infarction [22]. Furthermore, VDR has a wide spectrum of effects on various cells types implicated in atherosclerosis, including ECs [25–28], VSMCs [29–34], and immune cells [31, 35]. However, a causal relationship between reduced VDR expression and leukocyte-endothelial cell interplay leading to atherosclerosis has not yet been established. Here, we provide evidence for an important role of basal levels of VDR in limiting EC activation *in vitro* and offer a comprehensive study of signaling mechanisms by which these responses were elicited. Furthermore, we examined the effects of VDR deletion on atherosclerotic lesion formation in the apoE^-/-^ atherosclerosis model.

## Materials and Methods

### *In vitro* study

#### Cell culture and treatments

EA.hy926 cells (immortalized human vascular endothelial cell line) [36, 37] obtained from ATCC (CRL-2922) were cultured on 0.2% gelatin-coated tissue culture plates in DMEM media (BE-12-604F, Lonza, Barcelona, Spain) supplemented with 10% fetal bovine serum (FBS), penicillin and streptomycin. Fresh growth medium was changed every 2–3 days. Before treatments, cells were growth-arrested in serum-free medium and incubated separately with serum-free medium (control) or PS-1145 (10 μM) for indicated periods of time. Cells were maintained according to the described protocol, unless otherwise indicated. PMSF, protease inhibitor cocktail and PS-1145 were purchased from Sigma Chemical Co (Madrid, Spain).

#### Lentiviral production and infection of EA.hy926 cells

Plasmid to knockdown human VDR (clone ID: TRCN0000019507), with each hairpin sequence of the short hairpin RNA (shRNA) construct cloned into the lentiviral vector, pLKO.1, was purchased from Open Biosystems. Plasmid that expresses a super-repressor form of the NF-κB inhibitor IκBα was constructed as follows. Briefly, the insert of the plasmid pcDNA3-IκBα S32A/S36A [38] which expresses a super-repressor of NF-κB was subcloned via gateway technology into the lentiviral plasmid pDSL (ATTC) lacking the SV40-GFP cassette. Production of infective lentiviral particles was done as previously described [39]. Filtered supernatant was added to the growing culture of EA.hy926 cells and incubated overnight. Next day, fresh medium was replaced and the cells were left to grow for additional 2–3 days before starting puromycin selection (pLKO.1 constructs). The stable cell line was selected using 1 μg/ml puromycin selectable marker. Western blot was performed to check for VDR gene knockdown that was achieved after five passages.

#### Protein nuclear extraction

Cell monolayers were washed with cold PBS and scraped in Hypotonic buffer (20 mM Tris-HCl, pH 7.4, 10 mM NaCl, 3 mM MgCl_2_, 2 mM PMSF and protease inhibitor cocktail). After 15 minutes of incubation on ice, 15 μl of 10% Igepal/300 μl cell extract was added and vortexed for 10 sec at the highest setting. Homogenate was centrifuged at 10.000 x g for 15 minutes at 4°C and supernatant containing cytoplasmic fraction was stored at -80°C. Remaining cell pellet was further resuspended in Cell Extraction buffer (100 mM Tris, pH 7.4, 100 mM NaCl, 1% Triton X-100, 1 mM EDTA, 10% glycerol, 1 mM EGTA, 0.1% SDS, 0.5% deoxycholate, 20 mM Na_4_P_2_O_7_, 2 mM Na_3_VO_4_,1mMNaF, 2 mM PMSF and protease inhibitor cocktail) and incubated for 30 minutes on ice with vortexing at 10 minutes intervals. Obtained cell extract was centrifuged for 30 minutes at 14.000 x g, 4°C and supernatant containing nuclear fraction was stored at -80°C.

#### Western blot analysis

Total cell lysates were obtained by washing the cell monolayer with cold PBS, scraping and suspending in lysis buffer (125 mM Tris (pH 6.8), 2% SDS, 2 mM PMSF and protease inhibitor cocktail). 20 μg of proteins were electrophoresed on 8% or 10% SDS-PAGE gels, as previously described [39]. Positive immunoreactive bands were quantified by densitometry and compared with the expression of adequate loading control.

#### Leukocyte isolation

Human peripheral blood mononuclear cells (PBMC) were isolated as previously described [40]. The medical Ethical Committee of the Hospital Clínico Universitario de Valencia approved the study and all patients provided written informed consent.

#### Adhesion assay under flow conditions

Adhesion assay under flow conditions was done as previously described [41, 42]. Images were recorded in a single field of view over a 5 min period during which leukocyte parameters were determined. Leukocyte rolling was calculated by counting the number of leukocytes rolling over 100 μm^2^ of the endothelial monolayer during 1 min period. Velocities of 20 consecutive leukocytes in the field of focus were determined by measuring the time required to travel a distance of 100 μm. Leukocyte adhesion was determined by counting the number of leukocytes that maintained stable contact with the monolayer for 30 s.

### *In vivo* study

#### Generation of apoE-/-VDR-/- mice and experimental design

Mouse experiments performed in this study were approved by the Ethical Committee of the University of Lleida and carried out following the Guide for the Care and Use of Laboratory Animals published by the US National Institute of Health. VDR-deficient (VDR^-/-^) mice on a B6CBA genetic background [43], a kind gift from Dr. Kato (University of Tokyo, Japan), were backcrossed more than 8 times to C57BL/6J mice and have since been maintained in our colony for more than 7 years. To establish a line of apoE^-/-^VDR^-/-^ animals, apoE-deficient (apoE^-/-^) C57BL/6J mice (Jackson Laboratory, Bar Harbor, ME) were crossed with VDR^-/-^ mice to yield double-heterozygous progeny, which were intercrossed and the offspring was genotyped for VDR and apoE by PCR analysis of tail DNA. Mice obtained from these crossbreedings and used in our experiments had a mixed B6CBA background. All animals were weaned at 21 days. After weaning, apoE^-/-^mice were maintained on a regular mouse chow (Harlan Teklad, Madison, WI; USA), while apoE^-/-^VDR^-/-^ mice were fed a high-calcium, high-phosphate diet (rescue diet) (TD.96348, 20% Lactose, 2% Ca, 1.25% P; Harlan Teklad, Madison, WI; USA) to prevent hypocalcaemia. To induce atherosclerosis, apoE^-/-^ (n = 14) and apoE^-/-^VDR^-/-^ mice (n = 16) at 3 months of age were placed on a high fat-rescue diet (HFRD) (21% fat, 0.75% Cholesterol, 20% Lactose, 2% Ca, 1.25% P, S9358-E010, Ssniff, Soest, Germany) and drinking water with 1% Ca-gluconate and were kept for additional 2 months. All mice were housed and maintained in a barrier facility and pathogen-free procedures were employed in all mouse rooms. Animals were kept in a 12-hour light/dark cycle at 22°C with *ad libitum* access to food and water.

#### Genotyping of apoE and VDR by PCR

For genotyping the apoE gene, three primers, oIMR180, oIMR181, and oIMR182, were used as recommended by The Jackson Laboratory (Bar Harbor, ME). The sequences of the primers were as follows: oIMR180, 5’-GCC TAG CCG AGG GAG AGC CG-3’; oIMR181, 5’-TGT GAC TTG GGA GCT CTG CAG C-3’; oIMR182, 5’-GCC GCC CCG ACT GCA TCT-3’. Primer pair oIMR180 and oIMR181 was used to amplify a 155-bp wild-type band. Primer pair oIMR180 and oIMR182 was used to amplify a 245-bp band from the apoE-targeted allele. The PCR was carried out in a termocycler Techne TC-412 using the following program: 94°C, 5 min, 38 cycles of (94°C 30 sec, 62°C 30 sec, 72°C 30 sec), 72°C 10 min. For genotyping the VDR gene, the following primers were used: VDR071 (5’-ATG GAG GCA ATG GCA GCC AGC ACC TC-3’), VDR072 (5’-GAA ACC CTT GCA GCC TTC ACA GGT CA-3’), VDR073 (5’-GCC TGC TTG CGC AAT ATC ATG GTG GA-3’), VDR074 (5’-AGC CAG GTG AGT TTA CCT ACC ACT TCC-3’). Primer pair VDR071 and VDR072 amplified a 140-bp wild-type band, whereas primer pair VDR073 and VDR074 amplified a 450-bp Neo band from the inserted targeting vector, as described previously [44]. The following PCR program was used: 94°C, 5min; 35 cycles of (94°C 15 sec, 65°C 30 sec, 72°C 60 sec), 72°C 10 min.

#### Blood sampling and tissue collection

The apoE^-/-^ and apoE^-/-^VDR^-/-^ mice were euthanized at the age of 5 months. Blood was collected by cardiac puncture after a 16-hour overnight fast. The animals were perfused with PBS through a puncture in the left ventricle. Hearts and the upper thoracic aortas were rapidly removed, placed in cold PBS and subsequently cleaned of adherent fat and connective tissue under a dissecting microscope. Cleaned aortas were fixed in 4% paraformaldehyde overnight for the assessment of atherosclerotic lesions using *en face* technique. Hearts, fixed in 4% paraformaldehyde overnight, were embedded in paraffin for the assessment within the aortic root region. Down thoracic and whole abdominal aortas were snap-frozen in liquid nitrogen and stored at -80°C.

#### Quantification of atherosclerosis burden

Atherosclerosis was assessed by two approaches, first by the *en face* technique, and second, by cross-sectional assessment through the aortic root. For *en face* analysis, aorta was stained with Oil-Red O (0.2% Oil Red O in 80% MeOH) (Sigma Chemical Co, Madrid, Spain), opened longitudinally and pinned onto a black wax surface with micro needles. The extent of atherosclerosis on the mounted aorta was quantified by computer-assisted morphometric analysis (SigmaScan Pro5, Aspire Software International, Ashburn, Virginia), as previously explained [45]. The amount of aortic lesion formation in each animal was measured as percent lesion area per total area of the aorta. After *en face* analysis, aortic tissue was fixed again with 20% neutrally buffered formalin, embedded in paraffin, and cut at 5 μm-thick serial sections for immunohistochemical analysis. For the assessment of atherosclerosis in the valve area of the aortic root, serial cross-sections of heart (5-μm thick) were cut throughout the entire aortic root area. Sections were stained with hematoxylin/eosin and atherosclerosis was analyzed blindly in 5 cross sections of each specimen separated by 80 μm and covering 640 μm of the aortic root. Image analysis was performed with SigmaScan Pro5 software.

#### Immunohistochemical analysis of atherosclerotic plaques

Immunostaining for Mac-3, MCP-1 (Santa Cruz Biotechnology Inc., CA, USA) and αSMA (Sigma Chemical Co, Madrid, Spain) was carried out on 5-μm thick tissue sections of aortic arch and aortic root, as previously described [39]. Stained tissue sections were examined using a Nikon Eclipse 80i microscope with a Nikon automatic camera system. Immunohistochemical results of Mac-3 staining in the aortic arch region were evaluated following the uniform pre-established criteria. Staining intensity and % positive cells were graded semiquantitatively. Histological scores were obtained from each sample as follows: histoscore = 1X (% light staining) + 2X (% moderate staining) + 3X (% strong staining), which ranged from 0 (no immunoreaction) to 300 (maximum immunoreactivity). The reliability of such scores for interpretation of immunohistochemical staining of tissue sections has been shown previously [46, 47]. Immunohistochemical results of Mac-3 staining in the region of the aortic root was evaluated using SigmaScan Pro5 software and shown as percentage of stained area relative to the area occupied by atheroma.

For VCAM-1, ICAM-1(Santa Cruz Biotechnology Inc., CA, USA) and CD31 (Abcam, Cambridge, MA, USA), upon an overnight incubation with primary antibody at 4°C, sections were washed and the corresponding Alexa Fluor secondary antibodies (Life Technologies, Madrid, Spain) and Hoechst (Sigma Chemical Co, Madrid, Spain) were applied for one hour at RT. Quenching of possible autofluorescence was done by incubating the slides with 0.1% Sudan Black in 70% ethanol for 20 minutes in the dark followed by washing with PBS/0.02% Tween20. Sections were mounted with Slow Fade Reagent.

#### TUNEL assay

Apoptosis in the aortic arch region was determined using TUNEL, In Situ Cell Death Detection Kit (Promega, Madrid, Spain) as previously described [48].

#### Biochemical measurements

Serum calcium, phosphate, triglycerides and total, HDL and LDL cholesterol were determined by standard clinical methods using a multichannel Hitachi Modular analyzer (Roche Diagnostics, Indianapolis, USA). The method used for the analysis of serum calcium was the o-cresolphthalein complexone method and for serum inorganic phosphate was ammonium molybdate method. Serum levels of IL-6 and IL-6 from the cell supernatant were determined using the commercially available ELISAs kits from R&D Systems (Inc., MN, USA) or Diaclone SAS (Besancon, France), respectively.

#### Real time PCR

Total RNA was extracted from aorta using RNeasy Fibrous Tissue Mini Kit (Qiagen Iberia SL, Madrid, Spain) and from cultured cells using TRIzol reagent (Sigma Chemical Co, Madrid, Spain), following manufacturer’s instructions. Reverse transcription and real-time PCR was performed as previously described [39]. Briefly, real time PCR with gene-specific TaqMan probes for mouse VCAM-1 (Mm01320970_m1), ICAM-1 (Mm00516023_m1), IL-6 (Mm00446190_m1) (Applied Biosystems-Life Technologies S.A., Madrid, Spain) and human IL-6 (Hs.PT.58.40226675) (Integrated DNA Tech, Inc., Madrid, Spain) was performed with a CFX Real-Time PCR detection system (Bio-Rad Laboratories, S.A., Madrid, Spain) using TaqMan Universal PCR Master Mix, No AmpErase UNG. Forty cycles at 95°C for 15 seconds and 60°C for 1 minute were performed. Duplicate readings were taken, and the average was calculated. The relative mRNA levels were calculated by standard formulae (ΔΔCt method) using GAPDH (mouse, Mm99999915-g1 or human, Hs99999905-m1) as an endogenous control. The results referred to a randomly selected basal sample that was considered as value = 1.

#### Statistical analysis

The normality of the distribution was assessed by Kolmogorov Smirnov test. Statistical analysis was performed with GraphPad Prism 6.0. Differences between two groups were assessed by Student’s t test (unpaired or paired as needed depending on the study design). Differences between more than two groups were assessed by ANOVA, followed by the Tukey’s multiple comparison test. A *P*<0.05 was considered statistically significant. All data examined are expressed as mean ± SEM.

## Results

### Knockdown of VDR increases leukocyte-endothelial cell interactions and expression of markers of endothelial cell activation

**Fig 1:**
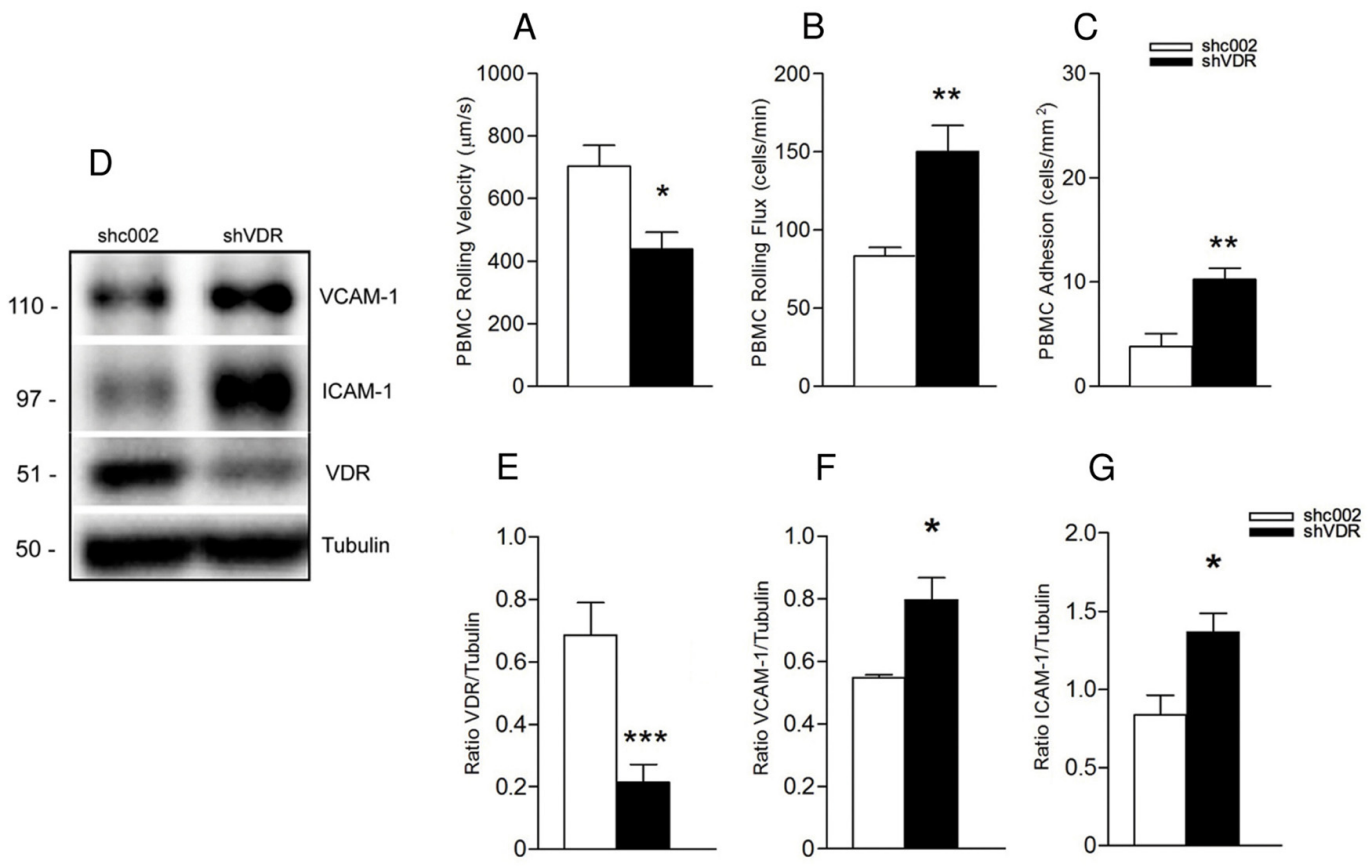
Knockdown of vitamin D receptor in endothelial cells leads to endothelial cell activation. Responses of leukocyte rolling velocity (A), rolling flux (B) and adhesion (C) to endothelial monolayer were monitored using 40x objective lens of an inverted microscope connected to a video camera. Data presented are mean ± SEM, n≥4, *p<0.05, **p<0.01 vs. corresponding shc002 cells. (D) Whole cell lysates were immunoblotted with antibodies against VCAM-1, ICAM-1 and VDR. The same samples were reprobed with tubulin to ensure equal loading. Representative Western blot (D) and quantitative analysis (E) demonstrate decrease in VDR expression in EA.hy926 infected with pLKO.1-VDR (shVDR) compared with pLKO.1-SHC002 (shc002 control). Data presented are mean ± SEM from 3 independent experiments, ***p<0.005 vs. shc002. Downregulation of VDR in endothelial cells resulted in increased VCAM-1 (D, F) and ICAM-1 (D, G) levels compared to control cells. *p<0.05 vs. control.

**Fig 2:**
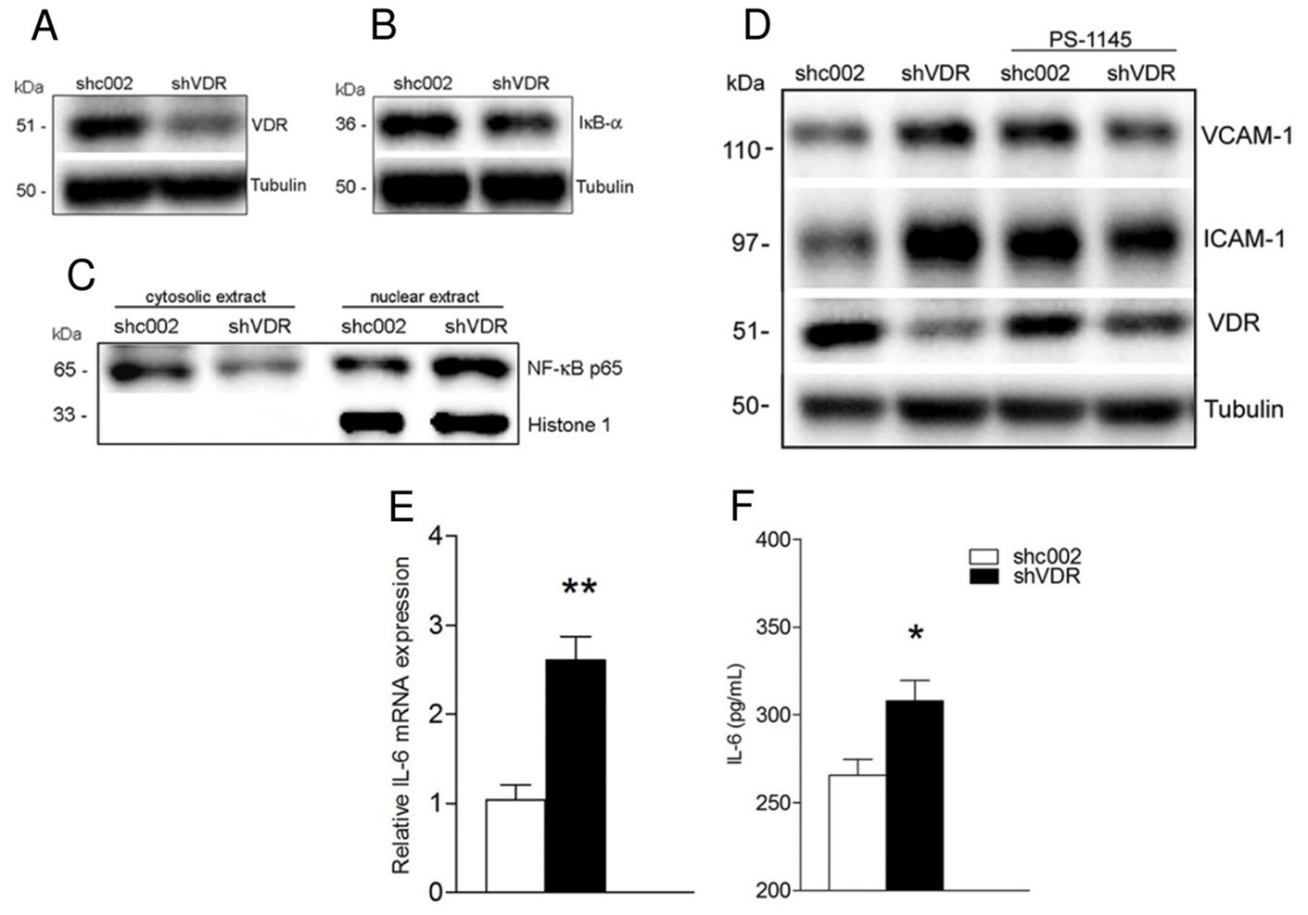
VDR knockdown-induced endothelial cell activation is mediated *via* NF-κB signaling. EA.hy926 cells (shc002 or shVDR) were untreated (A, B, C) or treated with IκB kinase (IKK) inhibitor PS-1145 (D) for 24 hours. (A, B, D) Whole cell lysates were immunoblotted with antibodies against VDR, IκB-α, VCAM-1 and ICAM-1. The same samples were reprobed with tubulin to ensure equal loading. (C) Western blot analysis of p65 levels in cytosolic and nuclear extracts isolated from shc002 and shVDR endothelial cells. The nuclear protein Histone 1, which is absent in the cytosolic fraction, served as a nuclear protein loading control. (D) Representative Western blot and quantitative analysis (G, H), *p<0.05 vs. shc002 or shVDR, respectivey (E) Real time PCR analysis of IL-6 mRNA in shc002 and shVDR endothelial cells. Data presented are mean ± SEM from 3 independent experiments. **p<0.01 vs. shc002. (F) Determination of IL-6 secretion into the medium by ELISA. Endothelial cells were grown in normal growth media for 72 hours. IL-6 production was determined using a human IL-6 HS ELISA kit. Data presented are mean ± SEM from 3 independent experiments. *p<0.05 vs. shc002.

To assess the role of VDR in leukocyte-endothelial interplay in basal conditions, the expression of VDR in endothelial cells was disrupted by shRNA delivery. Fig 1D shows an evident decrease of VDR expression in EA.hy926 cells infected with pLKO.1-VDR (shVDR) compared with pLKO.1-SHC002 (shc002 control). Leukocyte-endothelial interactions were evaluated using a well-established *in vitro* dynamic flow chamber system designed to visualize and analyze multistep recruitment of leukocytes in diseases such as atherosclerotic vascular disease [40]. Knockdown of VDR in endothelial cells (shVDR) led to endothelial cell activation, seen as a decrease in the rolling velocity of human PBMC over the endothelium (shc002: 704.2±66.54, shVDR: 438.04±55.29 μm/sec) (Fig 1A) and an increase in PBMC rolling flux (shc002: 83.25±5.74, shVDR: 150.3±16.59 cells/min) (Fig 1B) and adhesion (shc002: 3.78±1.28, shVDR: 10.23±1.11 cells/mm^2^) (Fig 1C) (S1 and S2 Videos). To further assess the role of VDR in endothelial cell activation, we analyzed the expression of vascular cell adhesion molecule-1 (VCAM-1) and intercellular adhesion molecule-1 (ICAM-1) in the same groups of cells. Knockdown of VDR in endothelial cells (shVDR) (Fig 1D and 1E) led to a significant increase of VCAM-1 (Fig 1D and 1F) and ICAM-1 (Fig 1D and 1G), compared with shc002 control. Furthermore, in our experimental model, downregulation of VDR led to a significant elevation of IL-6 mRNA, as well as of IL-6 secretion in shVDR cells compared with the corresponding controls (Fig 2E and 2F, respectively).

### VDR knockdown-induced endothelial cell activation is mediated *via* NF-κB signaling

Since NF-κB is a key regulator involved in the synthesis of adhesion molecules and inflammatory cytokines, we further assessed the protein levels of major components of the NF-κB pathway. The basal level of κB inhibitor (IκB)α protein, a key molecular target regulating NF-κB activation, was markedly decreased in shVDR cells compared with shc002 control (Fig 2B). Consistent with IκBα reduction, the basal level of p65 in the nuclear extracts was higher in shVDR cells than in controls (Fig 2C). The IκB kinase inhibitor, PS-1145, prevented upregulation of VCAM-1 and ICAM-1 in shVDR endothelial cells (Fig 2D, 2G and 2H), suggesting that upregulation of these adhesion molecules was mediated *via* NF-κB signaling.

**Fig 3:**
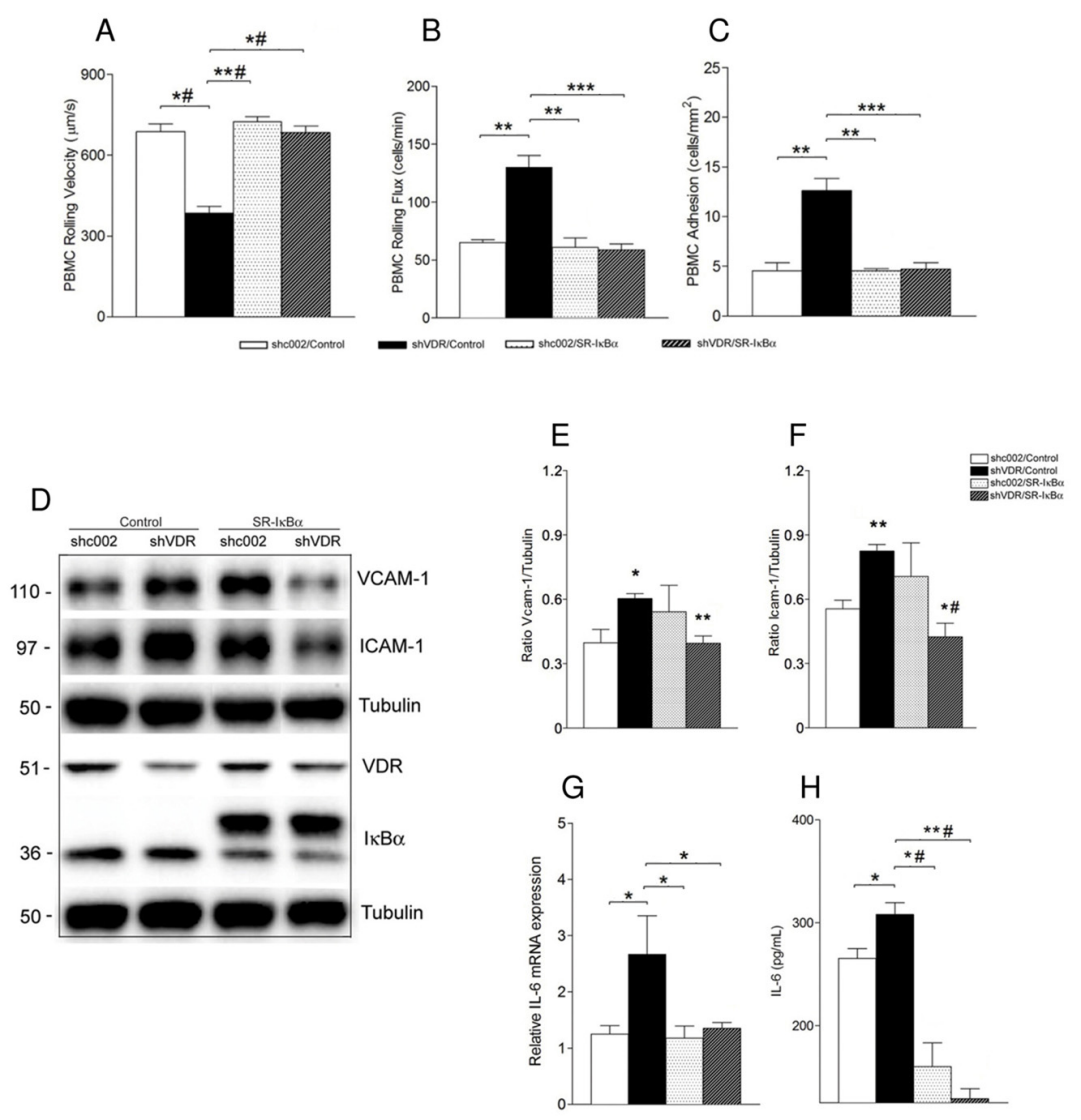
Super-repressor IκBα reduces VDR knockdown-induced endothelial cell activation. Responses of leukocyte rolling velocity (A), rolling flux (B) and adhesion (C) to endothelial monolayer were monitored using 40x objective lens of an inverted microscope connected to a video camera. Data presented are mean ± SEM, n≥4, **p<0.01, ***p<0.005, *#p<0.001. (D) Whole cell lysates were immunoblotted with antibodies against VCAM-1, ICAM-1, IκBα and VDR. The same samples were reprobed with tubulin to ensure equal loading. Representative Western blot (D) and quantitative analysis (E, F) *p<0.05 (VCAM-1) or **p<0.01 (ICAM-1) vs. shc002/Control; **p<0.01 (VCAM-1) or *#p<0.001 (ICAM-1) vs. shVDR/Control. (G) Real time PCR analysis of IL-6 mRNA. Data presented are mean ± SEM from 3 independent experiments. *p<0.05. (H) Determination of IL-6 secretion into the medium by ELISA. Endothelial cells were grown in normal growth media for 72 hours. IL-6 production was determined using a human IL-6 HS ELISA kit. Data presented are mean ± SEM from 3 independent experiments. *p<0.05, *#p<0.001, **#p<0.0001.

To corroborate the involvement of the NF-κB pathway in VDR knockdown-mediated endothelial cell activation, we stably overexpressed super-repressor IκBα in shc002 and shVDR cells. The super-repressor encodes an IκBα mutant protein, carrying serine-to-alanine mutations at amino acids 32 and 36, which prevent signal-promoted phosphorylation and subsequent degradation. It associates with and sequesters NF-κB in the cytoplasm, thereby preventing its nuclear translocation [49]. Inhibition of NF-κB activation with super-repressor IκBα significantly reduced VCAM-1, ICAM-1 (Fig 3D, 3E and 3F) and IL-6 (Fig 3G and 3H) in shVDR endothelial cells. The effects of disruption of the NF-κB axis were then examined in relation to VDR knockdown-mediated increase of leukocyte-endothelial cell interactions. As shown in Fig 3, VDR knockdown-induced decrease in the PBMC rolling velocity, as well as the increased PBMC rolling flux and adhesion to the endothelium were all markedly blunted in cells expressing IκBα mutant protein (Fig 3A, 3B and 3C).

### VDR deletion accelerates atherosclerotic plaque formation in apoE^-/-^ mice

**Fig 4:**
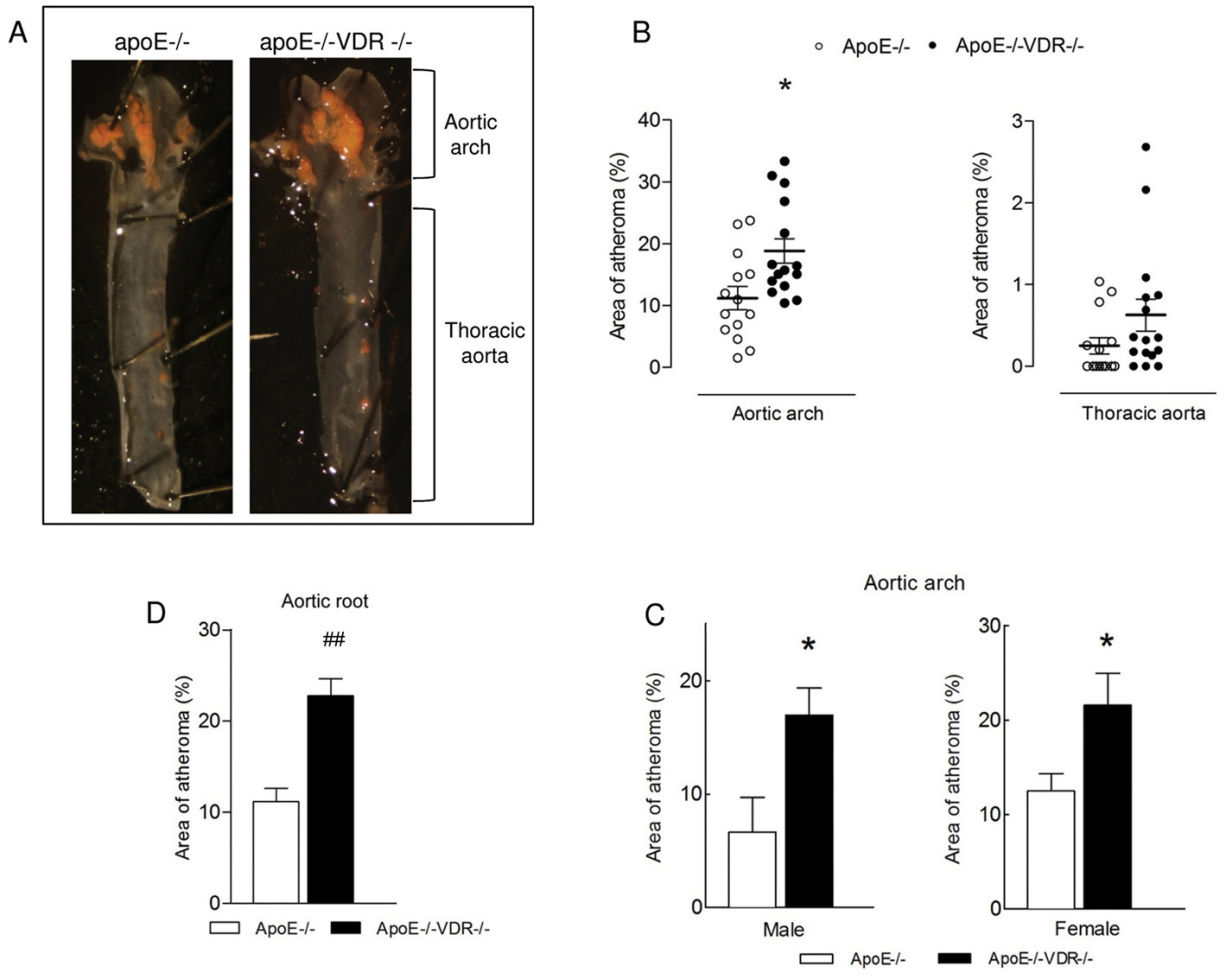
VDR deletion accelerates atherosclerosis in apoE^-/-^ mice. apoE^-/-^ and apoE^-/-^VDR^-/-^ mice were fed a high fat-rescue diet (HFRD) for 8 weeks. (A) Aortas were stained with Oil-Red O, opened longitudinally and pinned onto a black wax surface. Luminal side of the aorta is displayed, showing red lipid-rich atherosclerotic lesions within aortic arch and thoracic aorta. (B) Quantitative analysis of atherosclerotic lesion size within aortic arch and thoracic aorta. Data presented are mean ± SEM of 14–15 mice/group. *p<0.05 vs. apoE^-/-^. (C) Quantitative analysis of atherosclerotic lesion size within aortic arch in males and females of apoE^-/-^ and apoE^-/-^VDR^-/-^ mice. Data presented are mean ± SEM of 10 mice/group *p<0.05 vs. apoE^-/-^. (D) Quantitative analysis of atherosclerotic lesion size within aortic root. Five cross sections (spanning 640 μm of the aortic root) were analyzed per mouse, as explained in Methods. Data presented are mean ± SEM of n = 10 mice/group. ##p<0.0005.

To study the functional role of VDR in atherosclerosis, we generated animals lacking both VDR (VDR^-/-^) and apoE (apoE^-/-^) gene. Three months old apoE^-/-^VDR^-/-^ double knockout mice (DKO) and corresponding apoE^-/-^VDR^+/+^ mice (from now on termed apoE^-/-^) were fed a high fat rescue diet (HFRD) and drinking water containing 1% Ca-gluconate for 8 weeks. Atherosclerosis burden was quantified by planimetric analysis of whole-mounted oil Red-O-stained aortic arch and thoracic aorta and by analyzing cross-sections through the valve area of the aortic root. apoE^-/-^VDR^-/-^ mice demonstrated significantly enhanced atherosclerotic lesion formation in the aortic arch region (1.7-fold, p<0.0103), compared with apoE^-/-^mice (Fig 4A and 4B). The size of the plaque was also evaluated within each gender. Quantitative analysis showed significant increases in the lesion area of 2.5-fold in DKO males and 1.7-fold in DKO females compared with apoE^-/-^ counterparts (Fig 4C). In contrast, VDR deletion did not affect atheroma size in the thoracic aorta (Fig 4A and 4B). Consistent with the data from the *en face* method, cross-sectional examination of the valve area of the aortic root revealed that VDR deletion markedly accelerated atherosclerotic plaque formation in this highly susceptible region (2.04-fold, p<0,0002) (Fig 4D). Under the conditions of HFRD, both groups of mice maintained normal serum levels of calcium (8.8–10 mg/dL) and phosphate (5.5–6.01 mg/dL) (S1 Fig). Nevertheless, serum calcium concentrations of apoE^-/-^VDR^-/-^ mice were significantly lower than those of single apoE^-/-^ animals (S1 Fig).

### VDR deletion alters serum lipids levels

Elevated levels of serum lipids contribute significantly to atherosclerosis. Thus, we measured serum total, LDL and HDL cholesterol and triglycerides in apoE^-/-^ and apoE^-/-^VDR^-/-^mice following a 16-hour overnight fast. Both apoE^-/-^ and apoE^-/-^VDR^-/-^ mice developed marked hypercholesterolemia, with a similar lipid profile within each gender (S1 Fig). Nevertheless, total, LDL and HDL cholesterol levels were significantly lower in apoE^-/-^VDR^-/-^ mice than in apoE^-/-^ mice. Interestingly, after 2 months on a HFRD, apoE^-/-^VDR^-/-^ mice demonstrated practically normal levels of serum total triglycerides, compared with hypertriglyceridemic apoE^-/-^ mice (S1 Fig). The body weight of apoE^-/-^VDR^-/-^animals (males: 24.60±0.75 g; females: 19.33±0.99 g) was significantly reduced after the period of fat feeding, compared with apoE^-/-^ mice (males: 29.83±1.01 g; females: 22.38±0.92 g) (S2A and S2B Fig, respectively). However, apoE^-/-^VDR^-/-^ mice consumed more food than their apoE^-/-^ littermates (S2C Fig).

### VDR deletion affects atherosclerotic plaque composition

**Fig 5:**
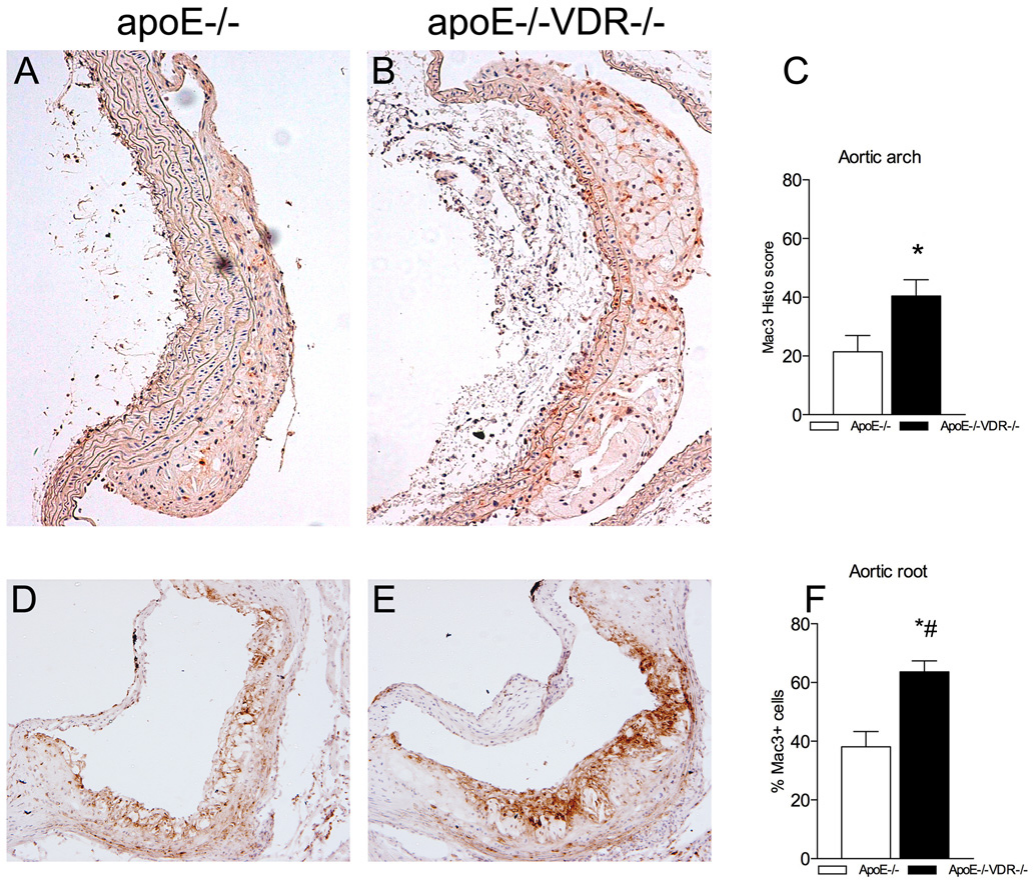
VDR ablation affects atheroma composition in apoE^-/-^ mice. Tissue sections of aortic arch (A, B) and aortic root (D, E) from apoE^-/-^ and apoE^-/-^VDR^-/-^ mice were stained with anti-Mac-3 to quantify plaque macrophage content. (C) Staining intensity and % of Mac-3 positive cells in the aortic arch were graded semiquantitatively and histological scores were obtained from every sample, as explained in Materials and Methods. (F) Quantification of Mac-3 staining in the aortic root is shown as percentage of stained area relative to the area occupied by atheroma. The photomicrographs (20x magnification) show representative images of aortic arch and aortic root sections. Differences between groups were evaluated by the Student t test. Data presented are mean ± SEM of 10 mice/group. *p<0.05, *#p<0.001 vs. apoE^-/-^ mice.

To determine how VDR affects the nature of plaques, we further investigated atherosclerotic plaque composition. The increased lesion areas of the aortic arch and aortic root of apoE^-/-^VDR^-/-^ mice correlated with increased macrophage (Mac-3) infiltration in both aortic regions (Fig 5A–5F). Immunoreactivity for monocyte chemoattractant protein (MCP-1), a proinflammatory chemokine important in monocyte recruitment into the vascular wall, was higher in lesions from apoE^-/-^VDR^-/-^ mice, while αSMA showed reduced immunoreactivity in the fibrous cap of DKO animals (S3A, S3B, S3C, S3D, S3G, S3H, S3I and S3J Fig). To assess the role of VDR in plaque’s apoptosis, aortic arch tissue cross-sections were examined using the terminal deoxynucleotidyl transferase dUTP nick-end labelling (TUNEL) method. We did not detect a difference in the number of TUNEL-positive cells in atheromas from apoE^-/-^VDR^-/-^ mice compared with apoE^-/-^ littermates (S3E and S3F Fig).

### Deletion of VDR leads to augmented expression of proinflammatory mediators *in vivo*

**Fig 6:**
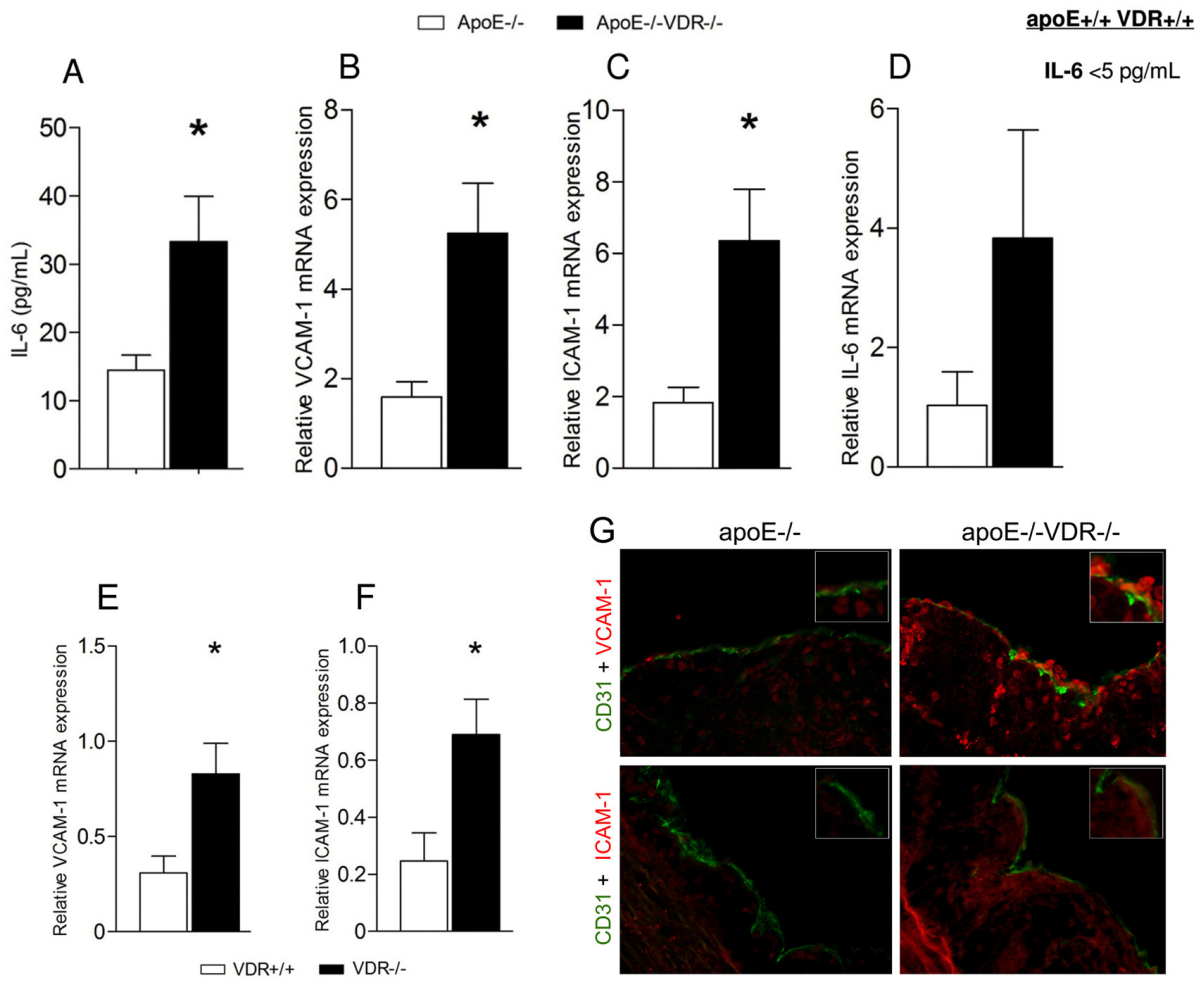
VDR deletion increases aortic and systemic inflammatory responses. (A) Effects of VDR deletion on proinflammatory cytokine production. Circulating serum levels of interleukin (IL-6) were determined by ELISA. Data presented are mean ± SEM of n = 10 mice/group. *p<0.05 vs. apoE^-/-^. (B, C, D, E, F) Real time PCR analysis of VCAM-1, ICAM-1 and IL-6 mRNA in the aortic tissue of apoE^-/-^ and apoE^-/-^VDR^-/-^ mice (B, C, D) and VDR^+/+^ and VDR^-/-^ mice (E, F). Data presented are mean ± SEM. (B, C) *p<0.05 vs. apoE^-/-^; (E, F) *p<0.05 vs. VDR^+/+^ mice. (G) Enhanced VCAM-1 and ICAM-1 expression in the vascular endothelium of apoE^-/-^VDR^-/-^ mice. Aortic root lesions from apoE^-/-^ and apoE^-/-^VDR^-/-^ mice were stained with anti-VCAM-1 and anti-ICAM-1. CD31 was used for endothelial cell staining. Representative images are shown in G. Magnification 20x.

After eight weeks on a HFRD diet, serum IL-6 levels were higher in apoE^-/-^VDR^-/-^ mice than in apoE^-/-^ control animals (Fig 6A). Furthermore, DKO mice exhibited markedly elevated expression of VCAM-1 (Fig 6B) and ICAM-1 (Fig 6C) in the aortae, as well as higher expression of IL-6 than apoE^-/-^ counterparts (Fig 6D). Immunohistochemical staining of the aortic root lesions showed increased expression of VCAM-1 protein, as well as a mild immunoreactivity for ICAM-1 in the vascular endothelium of DKO mice compared to apoE^-/-^ animals (Fig 6G). In the absence of the apoE-null genotype and evident lack of macrophage plaque accumulation, VDR^-/-^ mice still exhibited an increased arterial expression of adhesion molecules (Fig 6E and 6F).

## Discussion

It is now recognized that endothelial cell activation, in particular leukocyte recruitment and adhesion to the activated endothelium, plays an essential role in the initiation and progression of atherosclerosis. Vitamin D has been proposed to have an important role in cardioprotection, while its deficiency is a risk factor for the progression of cardiovascular damage. However, and although several pieces of information can partly explain this fact, the precise mechanisms by which defective vitamin D signaling increases cardiovascular mortality are still not fully understood. Thus, recent work from Schmidt et al.[50, 51] showed that VDR/vitamin D deficiency stimulates osteogenic key factor expression in the blood vessel wall increasing vascular calcification. Furthermore, a very recent paper from Ni *et al*. [27] has shown that elimination of VDR in endothelial cells caused endothelial dysfunction, a very early event in the development of atherosclerosis. In the current study, we demonstrated that impaired vascular signaling through VDR promoted endothelial cell activation *in vitro*. Furthermore, we showed that genetic deletion of VDR significantly increased atherosclerotic lesion formation in apoE^-/-^ mouse model.

Interactions of leukocytes with the endothelial cell layer of the arterial wall are early and critical events in acute and chronic inflammation, wound defense and repair [52]. However, dysregulation of leukocyte-endothelial interactions can lead to vascular dysfunction and injury associated with a plethora of vascular diseases such as atherosclerosis, diabetic vasculopathy, and hypertension [40, 53]. Indeed, an initial phase in the development of atherosclerotic lesion involves the recruitment of circulating inflammatory cells and their subsequent transendothelial migration [54, 55]. In our study, to assess the role of VDR in leukocyte-endothelial interactions, we used a widely acknowledged dynamic *in-vitro* experimental system that accurately reproduces some of the most important steps of the leukocyte recruitment cascade (rolling and adhesion) that anticipate the formation of an inflammatory focus *in vivo* [40]. In our experiments, downregulation of VDR in human endothelial cells induced a significant decrease in PBMC rolling velocity, while increasing rolling flux and adhesion of PBMC to the endothelium. These interactions of leukocytes with inflamed endothelial cells are predominantly mediated by a combination of cell surface adhesion molecules [54], among which vascular cell adhesion molecule 1 (VCAM-1) and intercellular cell adhesion molecule 1 (ICAM-1) emerged as the two leading molecules of the leukocyte recruitment cascade. VCAM-1 plays an important role in the slow rolling of monocytes along the surface of the endothelial layer, as well as in their firm adhesion [55]. ICAM-1, on the other hand, participates in adhesion strengthening, monocyte spreading and transendothelial migration [55, 56]. It is believed that by changing the expression levels of these adhesion molecules, endothelial cells modulate leukocyte adhesion and to some extent leukocyte rolling. In our study, VDR knockdown-mediated increase of leukocyte-endothelial cell interactions *in vitro* was accompanied by an upregulation of endothelial activation markers VCAM-1 and ICAM-1 in endothelial cells carrying shRNA targeting VDR. Furthermore, downregulation of VDR in endothelial cells led to a significant elevation of IL-6 secretion. IL-6 is known to promote endothelial cell activation and to increase expression of adhesion molecules [57], thereby perpetuating vascular inflammation [3]. Therefore, our results are in line with a previously published antibody-blocking study demonstrating that binding of lymphocytes to endothelial cells activated by IL-6 required VCAM-1 and ICAM-1 [57].

The effect of impaired VDR signalling on endothelial cell function and homeostasis *in vitro* seems to be related to activation of the NF-κB pathway. The NF-κB transcription factor upregulates inflammatory responses and has been widely implicated in the pathogenesis of atherosclerosis [58]. NF-κB is a family of transcription factors consisting of five members: p105/p50, p100/p52, p65 (RelA), RelB and c-Rel [59], that can form homo or heterodimers. In resting cells, NF-κB proteins are predominantly cytoplasmic associating with members of the inhibitory IκB family [59], that prevent NF-κB activation. Proinflammatory signals promote phosphorylation of IκB by IκB kinase (IKK) leading to IκB ubiquitination and subsequent proteasomal degradation [60]. The released NF-κB dimers enter the nucleus and activate the expression of many genes involved in the initiation and progression of atherosclerosis, including cytokines (e.g., TNF, IL-6), adhesion molecules (e.g., VCAM-1, ICAM-1) and chemokines (e.g., MCP-1) [58, 60]. Indeed, NF-κB was activated in macrophages, endothelial cells, and VSMCs in human and experimental atherosclerosis [60]. Our results suggest a direct involvement of VDR in prevention of NF-κB activation in endothelial cells. Namely, knockdown of VDR in endothelial cells led to IκBα downregulation, nuclear p65 accumulation, increased expression of IL-6 (a well-known NF-κB target gene), VCAM-1 and ICAM-1, alongside increased leukocyte-endothelial interactions, pointing to the existence of a causal relationship between the lack of VDR expression and endothelial cell inflammation. An increased activity of NF-κB has been reported in human osteoblasts [61], mouse embryonic fibroblast cells [62] and intestinal cells [63] lacking VDR. Furthermore, 1,25(OH)_2_D_3_ has been shown to reduce NF-κB expression and transcriptional activity [64–66], as well as the expression of many proinflammatory cytokines such as IL-1, IL-6, IL-8, and TNF-α in a variety of cell types [67, 68]. In our study, inhibition of the IκB kinase by PS-1145 prevented upregulation of VCAM-1 and ICAM-1 in shVDR endothelial cells, suggesting that upregulation of adhesion molecules in these cells was mediated *via* NF-κB signaling.

To substantiate the involvement of the canonical NF-κB pathway in VDR knockdown-mediated endothelial cell activation, we stably overexpressed a super-repressor IκBα in both shc002 and shVDR cells. Inhibition of NF-κB activation with super-repressor IκBα markedly blunted all signs of endothelial cell activation induced by VDR knockdown such as overexpression of VCAM-1, ICAM-1, IL-6, as well as leukocyte-endothelial cell interactions, adding a new piece of evidence regarding the involvement of the canonical NF-κB pathway.

To study the effects of VDR deletion on atherosclerotic plaque development *in vivo*, we combined a genetic model of VDR deletion with a mouse model of experimental atherosclerosis, the apolipoprotein E knockout (apoE^-/-^) mouse. We acknowledge that the model of the VDR KO is an absolute model that can give interesting information, but that cannot be compared to real situations of patients with a deficit of vitamin D. However, we demonstrated that deletion of VDR increased atherosclerotic lesion formation in apoE^-/-^VDR^-/-^ mice fed a HFRD. Furthermore, apoE^-/-^VDR^-/-^ mice showed significant changes in the lesion cellular content seen as an increase in the number of Mac-3^+^ macrophages in the aortic arch and aortic root regions, along with a higher immunoreactivity for MCP-1. There were no differences in TUNEL-positive apoptotic cells. Therefore, the enhanced plaque formation with abundant macrophage infiltration found in apoE^-/-^VDR^-/-^ mice could be due to enhanced leukocyte recruitment into the vascular wall. Owing to the nature of a total knockout mouse model, the possible effect of lacking VDR in other cell types, such as macrophages and VSMCs, should not be neglected. Previous studies have shown the role of vitamin D and the VDR in the process of atherosclerosis [35, 69]. In a very recent paper of Weng *et al*. [69], using mouse model of diet-induced vitamin D deficiency, the authors demonstrated the importance of vitamin D in the protection against atherosclerosis, highlighting vitamin D replacement as a potential therapy to attenuate this disease [69]. In accordance with our findings, Szeto *et al*. [35] showed that genetic lack of VDR led to a significant acceleration of plaque formation in LDLR^-/-^ mice, accompanied by increases in inflammatory molecules in the aorta and cholecterol influx in macrophages [35]. The authors pointed out to macrophage VDR signaling, specifically suppression of local RAS, as responsible for the inhibition of atherosclerosis in LDLR^-/-^ mice [35]. Our results, on the apoE^-/-^ model of atherosclerosis, agree with these previous reports and provide information on plaque size and composition in two highly susceptible areas for atherosclerosis, aortic arch and aortic root, *in vivo*. By dissecting the role of lacking VDR in endothelial cells *in vitro*, our data give a new perspective on the role of VDR in the atherogenesis process.

Interestingly, accelerated lesion formation in apoE^-/-^VDR^-/-^ mice was not correlated with serum lipid levels, known to be the major risk factor for atherosclerosis. Namely, apoE^-/-^VDR^-/-^ mice showed remarkably lower levels of total, LDL and HDL cholesterol than apoE^-/-^mice, alongside strikingly normal serum triglyceride levels, suggesting that lipid metabolism in apoE^-/-^ mice was affected by deletion of the VDR gene. Importantly, apoE^-/-^VDR^-/-^ mice were leaner, even though they consumed more food than their apoE^-/-^ counterparts. This phenotype is in line with previous studies on the phenotype of VDR^-/-^ mice describing markedly reduced body fat content after high fat feeding [70, 71]. Szeto *et al*. [35] observed similar effects of VDR deletion on lipid metabolism in LDLR^-/-^ mice, and proposed that the increased CYP7A1 activity could be responsible for the reduced plasma lipid levels in mice lacking VDR [35]. Another possible mechanism to explain lower lipid levels in apoE^-/-^VDR^-/-^ animals could be the regulation of uncoupling proteins (UCPs) expression in adipose tissue by vitamin D/VDR signaling [70, 72, 73]. Even though further studies should address the role of VDR in lipid metabolism, our current results suggest that the more severe atherosclerosis in apoE^-/-^VDR^-/-^ mice occurred despite improved serum lipid profiles.

*In vivo*, lack of vitamin D signaling led to an increased aortic expression of VCAM-1, ICAM-1 and IL-6 mRNA and higher immunoreactivity for adhesion molecules in the aortic root endothelium of DKO mice. Observed transcriptional differences in VCAM-1 and ICAM-1 may have come from endothelial cells, as well as other cells composing the blood vessel. Nevertheless, evidently higher immunoreactivity for VCAM-1 in the aortic root endothelium of DKO mice confirms the role for VDR in the vascular endothelium. Furthermore, we assessed changes in adhesion molecule expression in the aortas of VDR^+/+^ and VDR^-/-^ animals. The absence of dyslipidemia in these animals does not foster plaque growth, thus providing insights on proatherogenic mediators specifically associated with VDR deletion, without the direct effects of the proatherosclerotic (apoE^-/-^) genotype. Indeed, in the absence of atherosclerosis and macrophage accumulation, VDR ablation was still associated with an increased arterial expression of adhesion molecules implying that the enhanced plaque formation in apoE^-/-^VDR^-/-^ mice was due to a strong proinflammatory milieu fostered by VDR deletion. Expression of VCAM-1 and ICAM-1 has been consistently observed in atherosclerotic plaques. Several lines of evidence support an indispensable role of adhesion molecules in the development of atherosclerosis and plaque instability [54, 74]. We also observed remarkably higher levels of serum IL-6 in apoE^-/-^VDR^-/-^ mice. IL-6 has a pivotal role in atherosclerotic lesion progression and plaque stability [75, 76], and also regulates lipid metabolism [77, 78]. Short-term administration of excessive amounts of exogenous IL-6 led to an accelerated plaque development in young apoE^-/-^ mice [75]. Furthermore, genetic deficiency of IL-6 in apoE^-/-^ mice led to an enhanced plasma total cholesterol levels but decreased the accumulation of inflammatory cells in atherosclerotic plaques [79]. Administration of IL-6 to nonhuman primates [77] and cancer patients [78] resulted in a decrease of total cholesterol levels. Of note, in our study, apoE^-/-^VDR^-/-^ mice showed markedly higher levels of serum IL-6, along with enhanced aortic lesions and lower serum lipids. These results raise the possibility that upregulation of IL-6 *in vivo* in response to VDR deletion modifies two important aspects of atherosclerosis, lipid/lipoprotein levels and inflammatory/immune responses.

The results obtained in this study support the concept that constitutional VDR expression is required to prevent uncontrolled NF-κB activation in endothelial cells. Thus, impaired VDR signaling that led to endothelial cell activation and increased leukocyte-endothelium interactions might be one of the explanations for the more severe accumulation of inflammatory cells in atherosclerotic lesions. Nevertheless, the effect of the absence of VDR in other cell types, like macrophages and VSMCs, should not be disregarded. Data reported here provide novel information on the role of VDR signaling in endothelial cell homeostasis and in atherosclerosis.

## Supporting Information

S1 Fig Effects of VDR ablation on serum lipids, calcium and phosphate levels. apoE^-/-^ and apoE^-/-^VDR^-/-^ mice were fed HFRD for 8 weeks. Animals were fasted for 16 hours, blood was collected and serum total, LDL, HDL cholesterol, triglycerides, serum calcium and phosphate were determined as described in Methods. Data presented are mean ± SEM of 10 mice/group. *p<0.05, **p<0.01, *#p<0.001 vs. corresponding group of apoE^-/-^ mice.
(TIF)

S2 Fig Effects of VDR deletion on body weight and food intake in fat-fed apoE^-/-^ mice. (A, B) Body weight was measured at the beginning of the experiment (0 weeks) and at the end of fat feeding (8 weeks). apoE^-/-^VDR^-/-^ males (A) and females (B) exhibit lower body mass than their apoE^-/-^ counterparts after 8 weeks of fat feeding. (C) Individual food intake was measure after 2 months. apoE^-/-^VDR^-/-^ mice consumed more food than apoE^-/-^ animals. Data are mean ± SEM of 7–9 mice/group. *p<0.05; **p<0.01; *#p<0.001 vs. corresponding group of apoE^-/-^ mice.
(TIF)

S3 Fig Immunohistochemical staining for MCP-1, α-SMA and TUNEL assay. Representative photomicrographs of aortic arch (A-F) and aortic root sections (G-J) from apoE^-/-^ (A, C, E, G, I) and apoE^-/-^VDR^-/-^ mice (B, D, F, H, J) fed a HFRD for 8 weeks are presented. Sections were stained with anti-MCP-1 (A, B, G, H), anti- α-SMA (C, D, I, J) antibodies and TUNEL assay (E, F). MCP-1 showed higher immunoreactivity in the lesions of apoE^-/-^VDR^-/-^ mice compared with apoE^-/-^ counterparts. α-SMA showed reduced immunoreactivity in the fibrous cap of DKO mice compared with apoE^-/-^ animals. TUNEL analysis did not show differences in the number of apoptotic cells in the atherosclerotic lesions of two investigated groups of mice. (A, B, C, D, E, F) Original magnification x10; (G, H, I, J) Original magnification x20.
(TIF)

S1 Methods Additional information about the methods used in this manuscript can be found in this section. (DOC)

S1 Video Control cells. Video showing the adhesion under flow conditions of leukocytes to a monolayer of control endothelial cells.
(AVI)

S2 Video shRNA VDR cells. Video showing the adhesion under flow conditions of leukocytes to a monolayer of endothelial cells with a deletion of VDR protein.
(AVI)
