# Supplementary figures and images for: Impaired Vitamin D Signaling in Endothelial Cell Leads to an Enhanced Leukocyte-Endothelium Interplay: Implications for Atherosclerosis Development
Source: PLoS One. 2015 Aug 31;10(8):e0136863. doi: 10.1371/journal.pone.0136863 (PMC4556440; doi:10.1371/journal.pone.0136863)

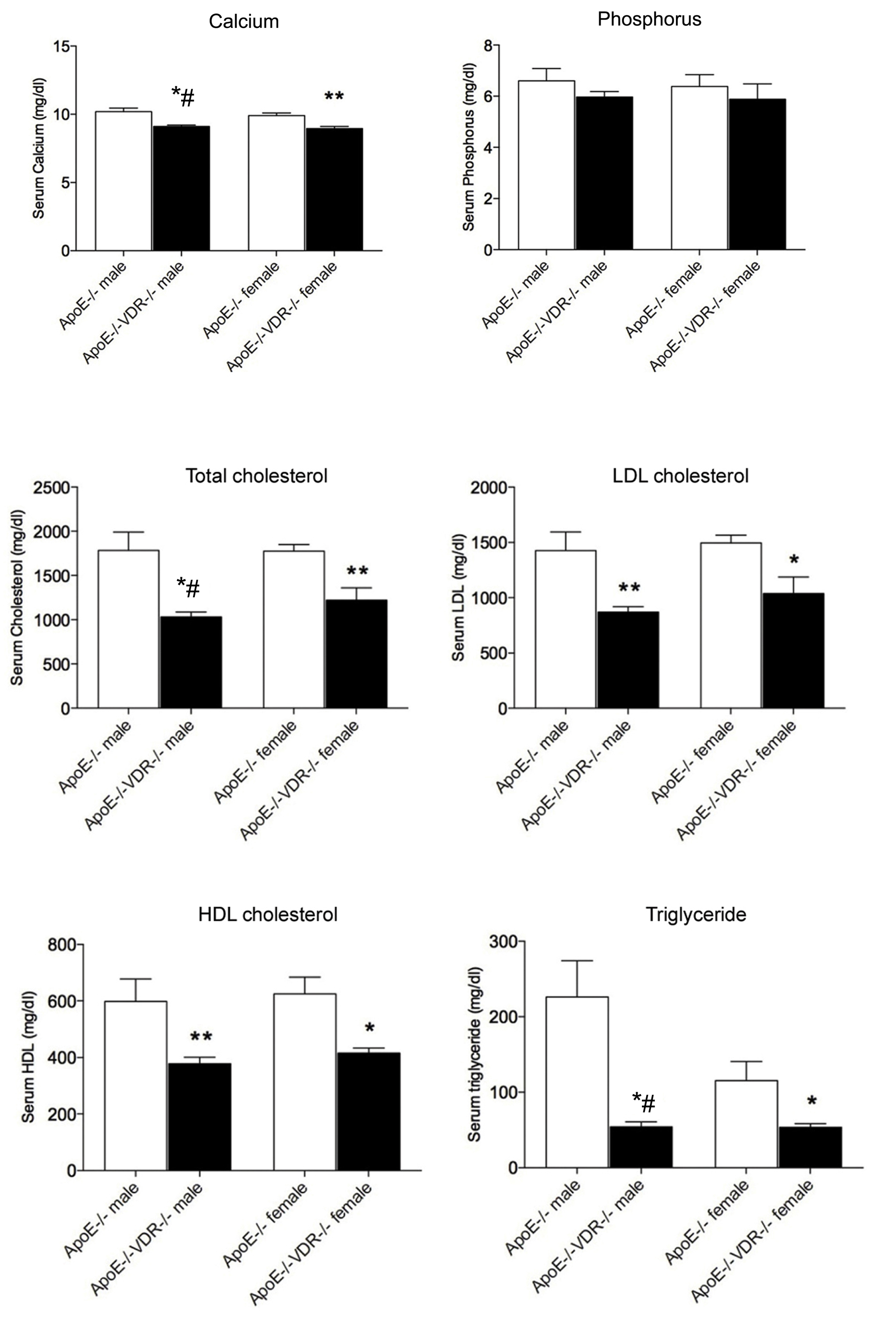

Supplement: S1 Fig — apoE-/- and apoE-/-VDR-/- mice were fed HFRD for 8 weeks. Animals were fasted for 16 hours, blood was collected and serum total, LDL, HDL cholesterol, triglycerides, serum calcium and phosphate were determined as described in Methods. Data presented are mean ± SEM of 10 mice/group. *p<0.05, **p<0.01, *#p<0.001 vs. corresponding group of apoE-/- mice. (TIF) [file pone.0136863.s001.tif]

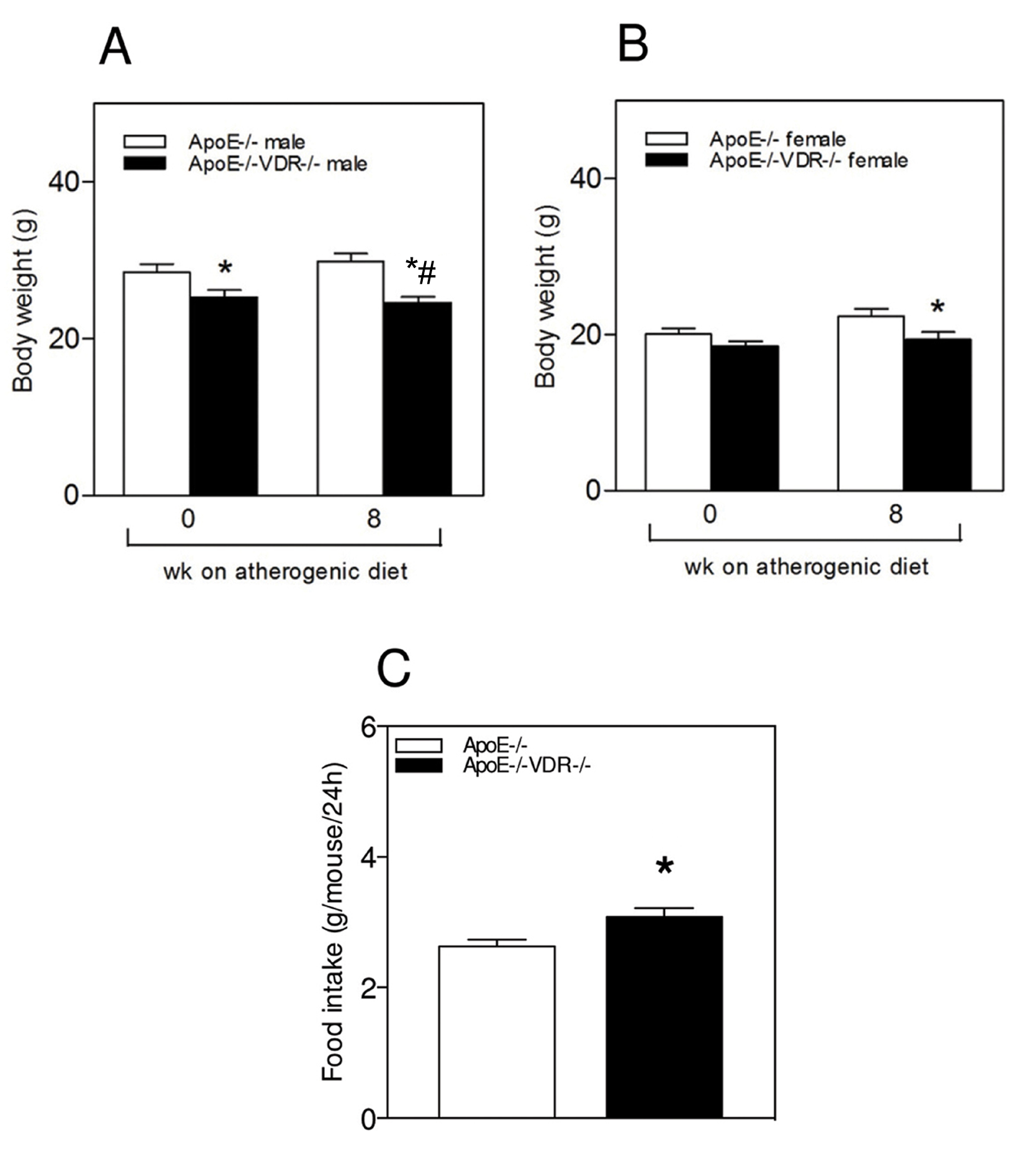

Supplement: S2 Fig — (A, B) Body weight was measured at the beginning of the experiment (0 weeks) and at the end of fat feeding (8 weeks). apoE-/-VDR-/- males (A) and females (B) exhibit lower body mass than their apoE-/- counterparts after 8 weeks of fat feeding. (C) Individual food intake was measure after 2 months. apoE-/-VDR-/- mice consumed more food than apoE-/- animals. Data are mean ± SEM of 7–9 mice/group. *p<0.05; **p<0.01; *#p<0.001 vs. corresponding group of apoE-/- mice. (TIF) [file pone.0136863.s002.tif]

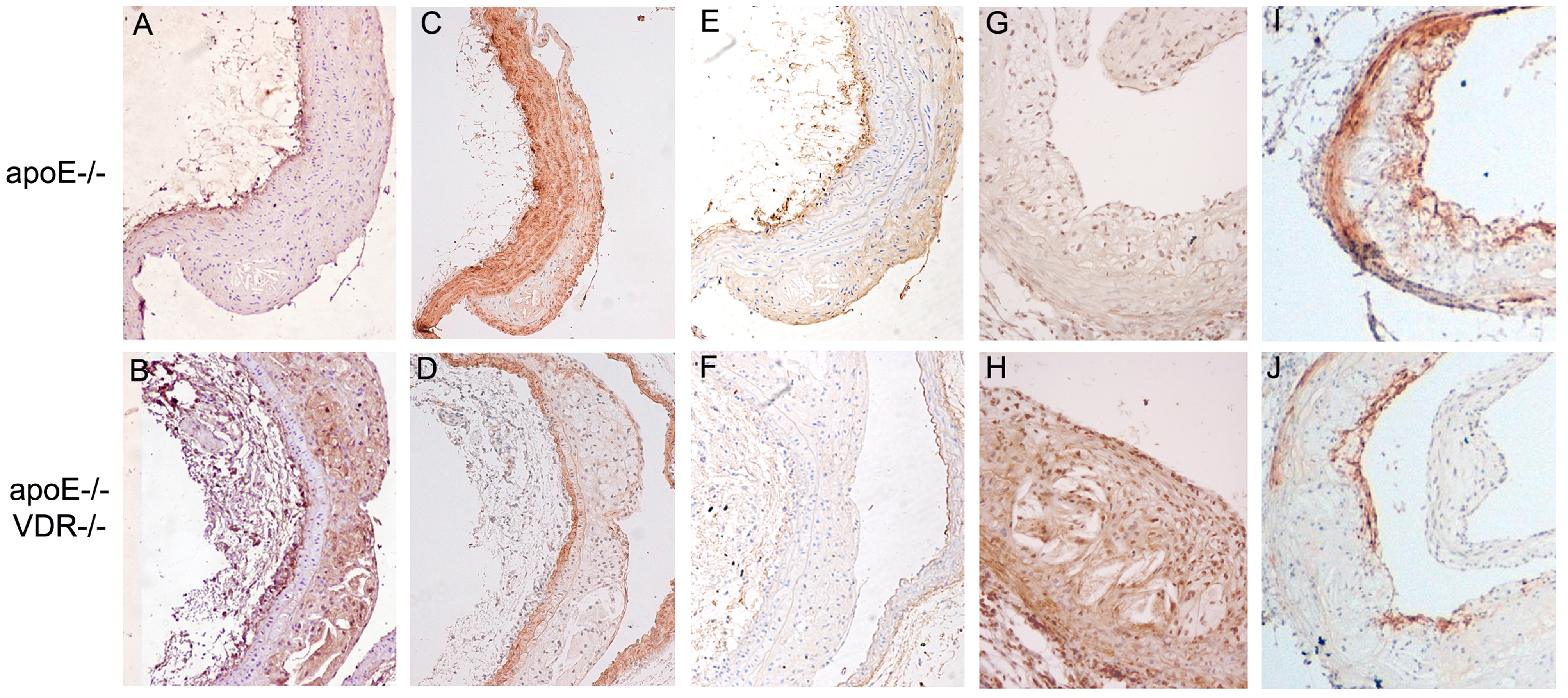

Supplement: S3 Fig — Representative photomicrographs of aortic arch (A-F) and aortic root sections (G-J) from apoE-/- (A, C, E, G, I) and apoE-/-VDR-/- mice (B, D, F, H, J) fed a HFRD for 8 weeks are presented. Sections were stained with anti-MCP-1 (A, B, G, H), anti- α-SMA (C, D, I, J) antibodies and TUNEL assay (E, F). MCP-1 showed higher immunoreactivity in the lesions of apoE-/-VDR-/- mice compared with apoE-/- counterparts. α-SMA showed reduced immunoreactivity in the fibrous cap of DKO mice compared with apoE-/- animals. TUNEL analysis did not show differences in the number of apoptotic cells in the atherosclerotic lesions of two investigated groups of mice. (A, B, C, D, E, F) Original magnification x10; (G, H, I, J) Original magnification x20. (TIF) [file pone.0136863.s003.tif]
