# Supplementary material for: Impaired Vitamin D Signaling in Endothelial Cell Leads to an Enhanced Leukocyte-Endothelium Interplay: Implications for Atherosclerosis Development
Source: PLoS One. 2015 Aug 31;10(8):e0136863. doi: 10.1371/journal.pone.0136863 (PMC4556440; doi:10.1371/journal.pone.0136863)
Supplement: S1 Methods — (DOC) [file pone.0136863.s004.doc]

**Supporting Information**

**Supplementary Methods**

Genotyping of apoE and VDR by PCR

For genotyping the apoE gene, three primers, oIMR180, oIMR181, and oIMR182, were used as recommended by The Jackson Laboratory (Bar Harbor, ME). The sequences of the primers were as follows: oIMR180, 5’-GCC TAG CCG AGG GAG AGC CG-3’; oIMR181, 5’-TGT GAC TTG GGA GCT CTG CAG C-3’; oIMR182, 5’-GCC GCC CCG ACT GCA TCT-3’. Primer pair oIMR180 and oIMR181 was used to amplify a 155-bp wild-type band. Primer pair oIMR180 and oIMR182 was used to amplify a 245-bp band from the apoE-targeted allele. The PCR was carried out in a termocycler Techne TC-412 using the following program: 94°C, 5 min, 38 cycles of (94°C 30 sec, 62°C 30 sec, 72°C 30 sec), 72°C 10 min. For genotyping the VDR gene, the following primers were used: VDR071 (5’-ATG GAG GCA ATG GCA GCC AGC ACC TC-3’), VDR072 (5’-GAA ACC CTT GCA GCC TTC ACA GGT CA-3’), VDR073 (5’-GCC TGC TTG CGC AAT ATC ATG GTG GA-3’), VDR074 (5’-AGC CAG GTG AGT TTA CCT ACC ACT TCC-3’). Primer pair VDR071 and VDR072 amplified a 140-bp wild-type band, whereas primer pair VDR073 and VDR074 amplified a 450-bp Neo band from the inserted targeting vector, as described previously^44^. The following PCR program was used: 94°C, 5min; 35 cycles of (94°C 15 sec, 65°C 30 sec, 72°C 60 sec), 72°C 10 min.
